# Prevalence and Risk Factors of Hernia in Patients With Rectus Abdominis Diastasis: A 10-Year Multicenter Retrospective Study

**DOI:** 10.3389/fsurg.2021.730875

**Published:** 2021-09-16

**Authors:** Sue Yuan, Honghong Wang, Jie Zhou

**Affiliations:** ^1^Xiangya Nursing School, Central South University, Changsha, China; ^2^Teaching and Research Section of Clinical Nursing, Xiangya Hospital of Central South University, Changsha, China; ^3^Department of Anesthesiology, Perioperative and Pain Medicine, Brigham and Women's Hospital, Harvard Medical School, Boston, MA, United States

**Keywords:** rectus abdominis diastasis, hernia, depressive disorder, chronic pain, risk factors

## Abstract

**Objectives:** Hernias are very common in patients with rectus abdominis diastasis (RAD). This study aimed to identify and compare the risk factors and patterns of hernia between men and women with RAD.

**Method:** We included patients with RAD from six hospitals within the Partners Healthcare System in Massachusetts, USA between 2009 and 2018. Univariate and multivariable binary logistic regression analyses were used to identify risk factors associated with hernia.

**Results:** Of the 1,294 RAD cases, 866 (67%) were women. The risk of RAD in women was 1.9 times greater than that of men. There were 240 men (56.1%) and 310 women (35.8%) having one or more hernia (*P* < 0.001). Of the 550 hernia cases, 278 men and 175 women had umbilical hernia (28.1 vs. 38.3%, *P* = 0.085). The distribution of hernia type differed between the two groups (*P* < 0.0001). Multivariate analysis identified that alcohol use [odd ratio (OR) 1.74 (1.17–2.59); *P* = 0.006] and depressive disorder [OR 1.90 (1.209–2.998); *P* = 0.005] were risk factors of coexisting hernia for men with RAD; age [OR 1.51 (1.33–1.72); *P* = 0.000] and smoking/tobacco use [OR 1.66 (1.13–2.44); *P* = 0.010] were risk factors of hernia for women.

**Conclusion:** The prevalence and risk factors of hernia in women with RAD significantly differed from that in men with RAD. Umbilical hernia is an important type of hernia. Alcohol use and depressive disorder in men, and age and smoking in women were risk factors of hernias in patients with RAD.

## Introduction

Rectus abdominis diastasis (RAD) is characterized by the thinning and widening of the *linea alba*, which is also defined as an interrectus distance of or more than 22 mm at 3 cm above the umbilicus when measured in a relaxed state (1). Patients usually present with a protruding midline following an increase in intraabdominal pressure. It is more common in obese middle-aged and older men and in women of reproductive age (2, 3). A high prevalence is found among women who underwent abdominal hysterectomies or hernia repair (38–52%) and among women during their third trimester of pregnancy (66–100%) (4–6). More attention has been drawn to RAD in the past decade. It was reported that patients with symptomatic umbilical and/or epigastric hernia all had concomitant RAD (7). Abdominal aortic aneurysm, muscular imbalance, low back pain, and lower extremity injury were all reported as coexisting symptoms of RAD. Among all, hernias were the most common (8–12).

Quality of life will decrease, and the burden of diseases will increase in patients living with RAD combined with hernia (13). To avoid the recurrence of RAD and hernia, numerous innovative and minimally invasive techniques were reported for the treatment of RAD with hernias, such as endoscopic-assisted *linea alba* reconstruction, liposuction abdominoplasty, and laparoscopic *linea alba* stapler repair (1, 7). Surgeons have also tried innovative suture techniques (such as the triangular mattress suture) or materials (such as smooth running absorbable polydioxanone suture, nylon, or polydioxanone) and/or have implemented different meshes (such as composite mesh, polypropylene mesh, or resorbable vinyl mesh) (1, 7, 13–15). However, the outcomes of these technical innovations are not very satisfactory, and the risk factors and patterns of concomitant hernia between men and women are unclear based on the current literature.

The incidence of RAD is increasing alongside the aging population and the growing obesity epidemic worldwide, providing a greater insight into the characteristics of this special population. Therefore, we conducted this retrospective study to explore the characteristics of hernia in patients with RAD. We hypothesized that the prevalence, patterns, and risk factors of hernia in women with RAD differ from that in men with RAD.

## Materials and Methods

### Study Design and Data Source

This retrospective study analyzed coexisting conditions of patients with RAD from six hospitals within the Partners Healthcare System in Massachusetts, USA. The institutional review board of the research of Partners approved the study protocol. Patient data were extracted from Partners research patient data registry (RPDR). Medical records of patients with a diagnosis of RAD (International Classification of Diseases, Ninth Revision Clinical Modification, ICD-9-CM: 728.84; ICD-10-CM: M62.08) from the hospitals of the Partners HealthCare System between January 1, 2009 to December 31, 2018 were reviewed. Individual-level data including diagnosis, primary source ultrasound (when available), demographic information, health history, open surgery history (open abdominal operations were included, but laparoscopic operations were excluded), operative notes, imaging reports, and procedures were extracted from RPDR.

### Data Collection

The demographic and health history variables included in this study were age, race or ethnicity, body mass index (BMI), abdominal surgery history, smoking status, and alcohol use. Except for hernia, other coexisting conditions of the patients were identified and they included low back pain; pelvic and perineal pain; incontinence; strain of muscle, fascial, and tendon (SMFT); and depressive disorder. The diagnoses above were defined by ICD-9-CM and ICD-10-CM diagnoses codes. We reviewed the trend of hernia. The cumulative composition ratio of hernia was defined as the percentage of patients who were diagnosed with the disease in the past or current year to account for all patients. Age at the onset of the disease was defined as the age for the first time when the patients were diagnosed with this disease. Abdominal wall hernia, in this study, mainly included incisional hernias (which occur along incisions from a prior surgery), diaphragmatic hernias, ventral hernias, inguinal hernias (femoral hernias), and umbilical hernias, unilaterally or bilaterally, referring to the definition of abdominal wall hernia (16).

### Statistical Analyses

All statistical analyses were performed using SPSS Statistics version 20 (IBM Corp., Armonk, NY, USA). Data are summarized as mean and standard deviation or frequencies and percentages where applicable. Pearson's and continuity correction chi-square test was used to test the differences between groups for counting data and Wilcoxon's *W* non-parametric test was used for continuous variables with non-normal distribution. Univariate and multivariable binary logistic regression analyses were used to identify the independent risk factors of hernia. The odd ratio (OR) and 95% confidence interval (CI) were estimated from the multivariate regression analysis. To reduce confounding bias, we adopted a forward stepwise regression analysis (α entry = 0.10, α removal = 0.10). A *P* < 0.05 was considered statistically significant; *P*-values were two-sided except for two that were one-sided. Risk ratio formulation was used to calculate the relative risk. Missing data was treated as system-missing values automatically.

## Results

### Prevalence of Concomitant Hernia in Patients With RAD

**Figure 1:**
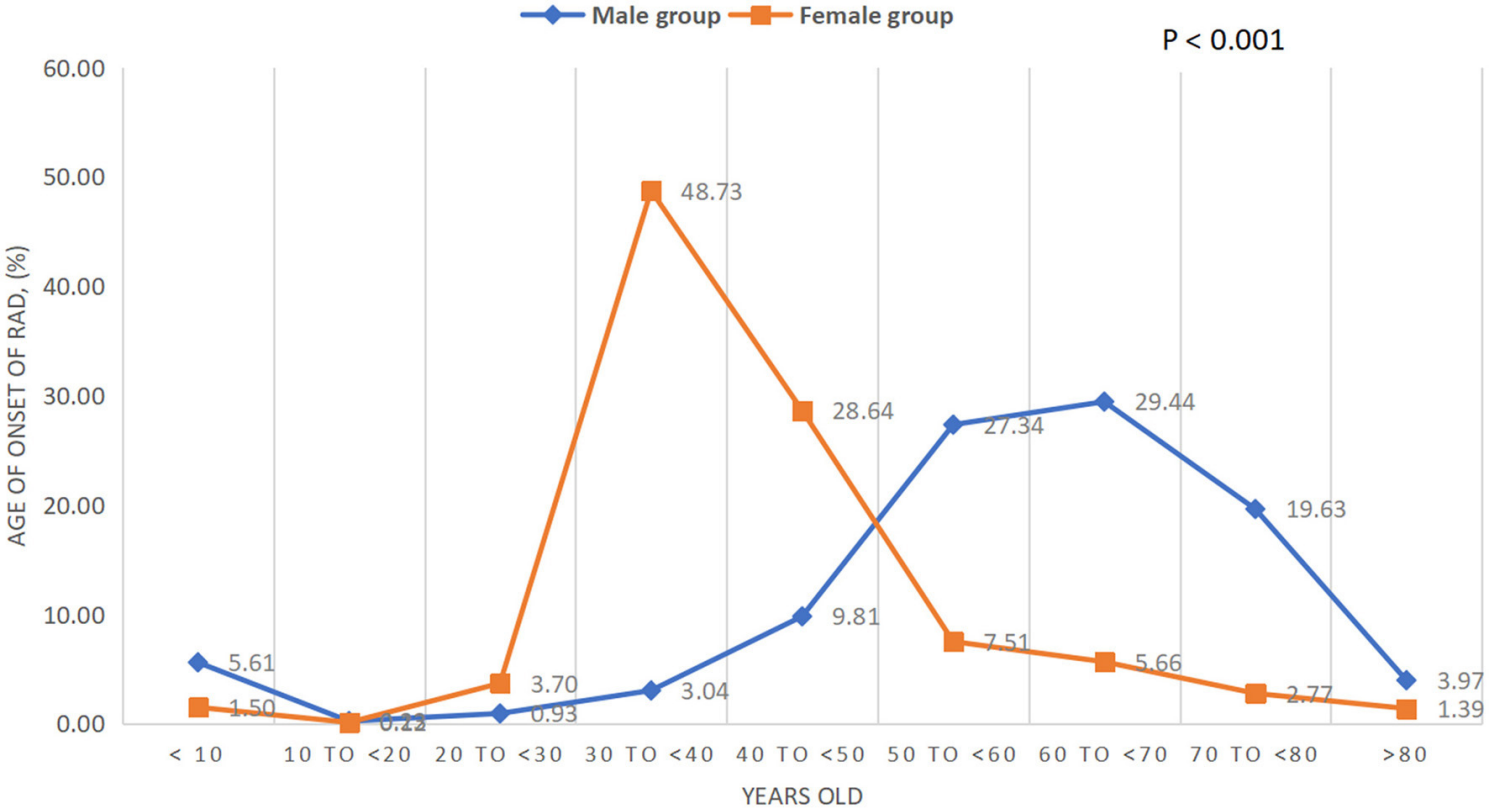
Age at onset of RAD.

**Figure 2:**
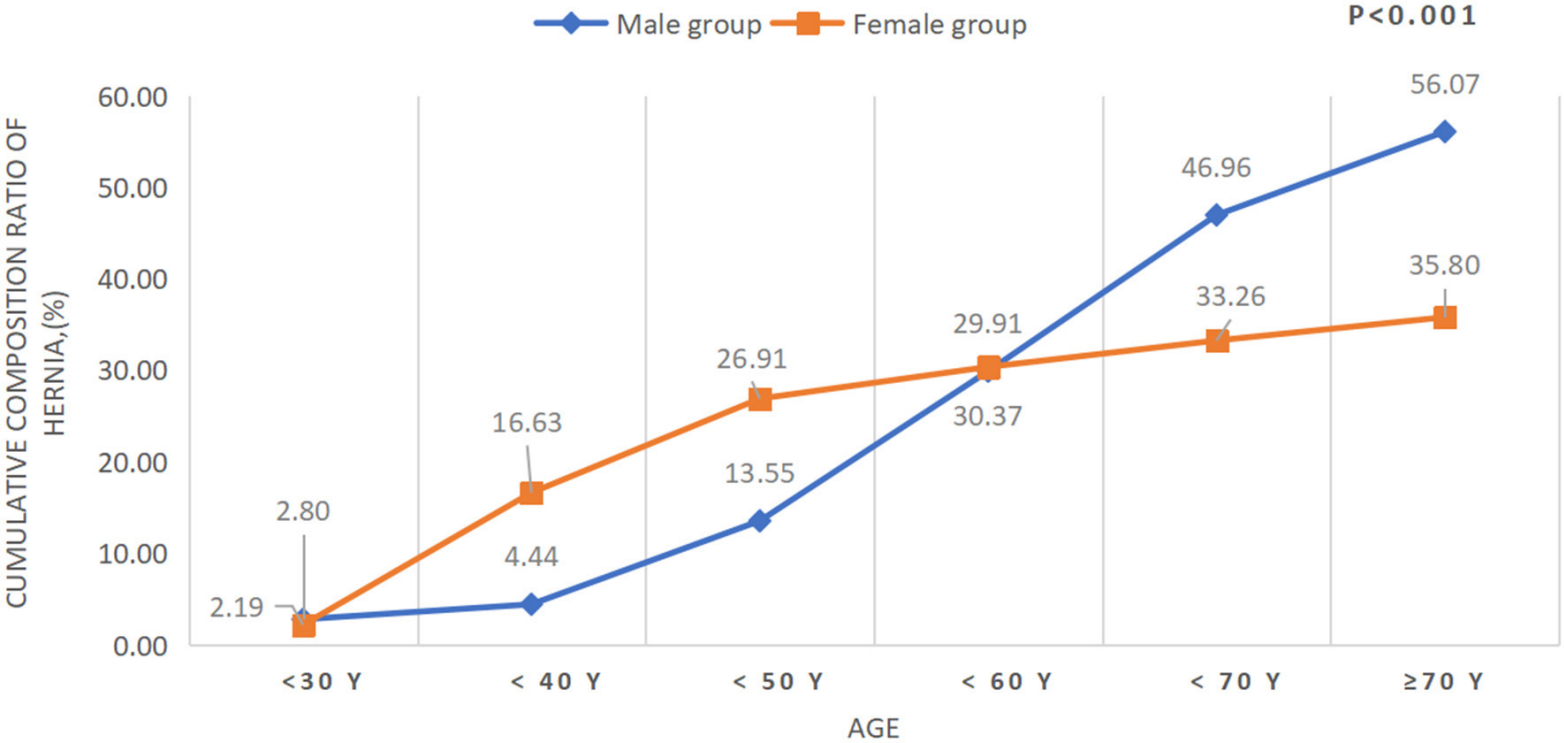
Cumulative composition ratio of hernia.

Clinical characteristics, including demographics, social history, abdominal surgery history, and coexisting conditions at presentation, are outlined for all patients (Table 1). Of the 1,294 cases, 866 were women (66.9%) and 313 had a history of one or more open abdominal surgery (24.2%). Female subjects demonstrated a 1.9 times greater risk of developing RAD than male subjects in this geographical area. It is worth noting that women account for 51.5% of the population in Massachusetts (17). The mean age at the onset of RAD in the male group was significantly higher than the female group (41 vs. 64 years old, *P* < 0.001), and the age in the female group was concentrated in the 30–40 years old age group, which was earlier than that in the male group (50–70 years old age group) (*P* for trend <0.001, Table 1 and Figure 1). The mean BMI in the female group was significantly lower than that of the male group (28.6 ± 6.8 kg/m^2^ vs. 31.9 ± 5.0 kg/m^2^, *P* < 0.0001, Table 1). Of the 1,294 cases, 37 patients were younger than 10 years and 24 of them were men (64.9%). The top three concomitant disorders with RAD we identified were hernia (42.5%), low back pain (28.0%), and depressive disorders (26.2%) (Table 1). Both smoking/tobacco and alcohol use in the male group were significantly higher than that in the female group (35.0 vs. 16.3%, *P* < 0.0001; 44.4 vs. 37.6%, *P* = 0.0022; respectively). Of the 1,294 cases, 550 of them (42.5%) had one or more hernia with a total of 823 hernia, 366 for men and 457 for women. The most common types of hernia (unilateral or bilateral) in patients with RAD were ventral hernia (35.8%) for men and umbilical hernia (38.4%) for women, and the distribution of hernia type differed between the two groups (*P* < 0.0001, Table 1). There were 278 subjects (33.8%) having umbilical hernia in this study, 103 of them in the male group and 175 in the female group (28.1 vs. 38.3%, *P* = 0.085). The cumulative composition ratio of hernia in women before the age of 60 was higher than in men; however, this trend begins to rapidly increase in men over the age of 60 (*P* for trend <0.001, Table 1 and Figure 2).

**Table 1:** Characteristics of patients with RAD.

**Characteristics**	**No. (%)**			***P-*value**
	**Male group**	**Female group**	**Total**	
	**(*n* = 428)**	**(*n* = 866)**	**(*n* = 1,294)**	
**Demographic characteristics**
**Age (the age at onset of RAD, y)**
Median (Percentiles), y	64 (56–71)	41 (36–52)	51 (39–65)	<0.001^†^
<30	28 (6.5)	31 (3.6)	59 (4.6)	<0.001
30 to <40	12 (2.8)	362 (41.8)	374 (28.9)	
40 to <50	36 (8.4)	309 (35.7)	345 (26.6)	
50 to <60	113 (26.4)	68 (7.9)	181 (14.0)	
60 to <70	127 (29.7)	54 (6.2)	181 (14.0)	
≥70	112 (26.2)	42 (4.8)	154 (11.9)	
**Race or ethnic group**
Non-Hispanic Black	20 (4.7)	42 (4.8)	62 (4.8)	0.964
Hispanic	19 (4.4)	36 (4.2)	55 (4.3)	
Other	389 (90.9)	788 (91.0)	1177 (91.0)	
**Body mass index**
Mean (kg/m^2^)	31.9 ± 5.0	28.6 ± 6.8	29.9 ± 6.4	<0.001^†^
Category				
<18.5	13 (3.1)	14 (1.7)	27 (2.1)	<0.001
18.5 to <25	30 (7.2)	304 (35.9)	334 (26.4)	
25 to <30	115 (27.4)	263 (31.1)	378 (29.9)	
30 to <40	230 (54.9)	217 (25.6)	447 (35.3)	
≥40	31 (7.4)	49 (5.8)	80 (6.3)	
Missing data	9 (2.1)	19 (2.2)	28 (2.2)	
**Abdominal surgery history (before the age at onset of RAD)**	48 (11.2)	265 (30.6)	313 (24.2)	
**Social history**
**Smoking/tobacco use**	150 (35.0)	141 (16.3)	291 (22.5)	<0.001
**Alcohol use**	190 (44.4)	326 (37.6)	516 (39.9)	0.022
**Depressive disorder**	116 (27.1)	223 (25.8)	339 (26.2)	0.638
**Major depressive disorder**	84 (19.6)	152 (17.6)	236 (18.2)	0.360
**Coexisting conditions**
**Hernia**	240 (56.1)	310 (35.8)	550 (42.5)	<0.001
Diaphragmatic hernia	42 (11.5)	46 (10.1)	88 (10.7)	<0.001
Ventral hernia	131 (35.8)	167 (36.5)	298 (36.2)	
Inguinal hernia	50 (13.7)	21 (4.6)	71 (8.6)	
Incisional hernia	40 (10.9)	48 (10.5)	88 (10.7)	
Umbilical hernia	103 (28.1)	175 (38.3)	278 (33.8)	
**Low back pain**	151 (35.3)	211 (24.4)	362 (28.0)	<0.001
**Pelvic and perineal pain**	43 (10.0)	144 (16.6)	187 (14.5)	0.001
**Incontinence**	8 (1.9)	35 (4.0)	43 (3.3)	0.040
**Strain of muscle, fascial, and tendon**	50 (11.7)	77 (8.9)	127 (9.8)	0.113
Strain of muscle, fascial and tendon of lower back	7 (1.6)	14 (1.6)	21 (1.6)	1

† *Wilcoxon W non-parametric test*.

### Patterns of Concomitant Hernia With BMI

Tables 2, 3 show the prevalence of concomitant hernias and social history that were evaluated using BMI = 30 kg/m^2^ as the cutoff value. For the women in this study, patients with BMI of 30 kg/m^2^ or higher had higher concomitant hernias (45.5 vs. 31.8%, *P* < 0.001), especially diaphragmatic hernia (11.3 vs. 2.8%, *P* < 0.001), ventral hernias (28.9 vs. 15.3%, *P* < 0.001), and incisional hernia (8.3 vs. 4.3%, *P* = 0.019). Women with BMI of 30 kg/m^2^ or higher had higher concomitant depressive disorder (39.5 vs. 20.2%, *P* < 0.001) and major depressive disorder (27.1 vs. 13.7%, *P* < 0.001) as well (Table 3). There was no difference in the prevalence of hernias in the male group when compared with the two groups based on BMI category. Significantly differences in terms of smoking statuses were noted in both the male (39.5 vs. 29.7%, *P* = 0.046) and the female group (24.1 vs. 13.2%, *P* < 0.001, Table 2).

**Table 2:** Characteristics of RAD men depending on BMI category.

**Characteristics**	**No. (%)**				***P***
	**BMI** ** <30 (** ***N*** **=** **158)**		**BMI** **≥30 (** ***N*** **=** **261)**		
	**Yes**	**No**	**Yes**	**No**	
**Coexisting hernia**
Hernia	84 (53.2)	74 (46.8)	154 (59.0)	107 (41.0)	0.242
Diaphragmatic hernia	11 (7.0)	147 (93.0)	31 (11.9)	230 (88.1)	0.104
Ventral hernia	46 (29.1)	112 (70.9)	85 (32.6)	176 (67.4)	0.460
Inguinal hernia	19 (12.0)	139 (88.0)	31 (11.9)	230 (88.1)	0.964
Incisional hernia	15 (9.5)	143 (90.5)	25 (9.6)	236 (90.4)	0.977
Umbilical hernia	31 (19.6)	127 (80.4)	70 (26.8)	191 (73.2)	0.095
**Social history**
Smoking/tobacco use	47 (29.7)	111 (70.3)	103 (39.5)	158 (60.5)	0.044
Alcohol use	67 (42.4)	91 (57.6)	123 (47.1)	138 (52.9)	0.347
Depressive disorder	39 (24.7)	119 (75.3)	77 (29.5)	184 (70.5)	0.285
Major depressive disorder	28 (17.7)	130 (82.3)	56 (21.5)	205 (78.5)	0.355

**Table 3:** Characteristics of RAD women depending on BMI category.

**Characteristics**	**No. (%)**				***P***
	**BMI** ** <30 (** ***N*** **=** **581)**		**BMI** **≥30 (** ***N*** **=** **266)**		
	**Yes**	**No**	**Yes**	**No**	
**Coexisting hernia**
Hernia	185 (31.8)	396 (68.2)	121 (45.5)	145 (54.5)	<0.001
Diaphragmatic hernia	16 (2.8)	565 (97.2)	30 (11.3)	236 (88.7)	<0.001
Ventral hernia	89 (15.3)	492 (84.7)	77 (28.9)	189 (71.1)	<0.001
Inguinal hernia	15 (2.6)	566 (97.4)	6 (2.3)	260 (97.7)	0.777
Incisional hernia	25 (4.3)	556 (95.7)	22 (8.3)	244 (91.7)	0.019
Umbilical hernia	117 (20.1)	464 (79.9)	55 (20.7)	211 (79.3)	0.856
**Social history**
Smoking/tobacco use	77 (13.2)	507 (86.8)	64 (24.1)	202 (75.9)	<0.001
Alcohol use	219 (37.5)	365 (62.5)	107 (40.2)	159 (59.8)	0.449
Depressive disorder	118 (20.2)	466 (79.8)	105 (39.5)	161 (60.5)	<0.001
Major depressive disorder	80 (13.7)	504 (86.3)	72 (27.1)	194 (72.9)	<0.001

*(1) One ventral hernia cases with missing BMI value*.

### Risk Factors of Hernia

Based on gender classification, we further categorized these data into two subgroups depending on the presence of the concomitant hernia. The mean age in the hernia subgroup was significantly higher than that in the non-hernia subgroup, both in the male group (6.5 ± 15.4 years old vs. 56.3 ± 2.5 years old, *P* = 0.028 [1-tailed], Table 4) and in the female group (46.9 ± 13.8 years old vs. 4.6 ± 11.1 years old, *P* < 0.001, Table 5). Women with concomitant hernia had significantly higher mean BMI compared to those without hernia (29.2 ± 7.1 kg/m^2^ vs. 27.4 ± 6.3 kg/m^2^, *P* = 0.001, Table 5). Univariate and multivariate binary logistic regression analysis predicted coexisting hernia occurrence in patients with RAD. For men with RAD, multivariate analysis identified that alcohol use (OR, 1.74; 95% CI, 1.17–2.59; *P* = 0.006) and depressive disorder (OR, 1.90; 95% CI, 1.209–2.998; *P* = 0.005) were risk factors of coexisting hernia (Table 4); for women with RAD, age (OR, 1.51; 95% CI, 1.33–1.72; *P* = 0.000) and smoking/tobacco use (OR, 1.66; 95% CI, 1.13–2.44; *P* = 0.010) were risk factors of coexisting hernia (Table 5).

**Table 4:** Characteristics of RAD men with or without hernia.

**Variable**	**No. (%)**		**Univariate**		**Multivariate**	
	**Non-hernia subgroup (*N* = 188)**	**Hernia subgroup (*N* = 240)**	***P*-value**	**OR (95% CI)**	***P*-value**	**OR (95% CI)**
**DEMOGRAPHIC**
Age, mean (*SD*), y	56.3 ± 20.5	60.5 ± 15.4	0.028 (1–tailed)^†^			
<30	18 (9.6)	10 (4.2)	0.128	1	0.107	NS
30 to <40	8 (4.3)	4 (1.7)	0.885	0.90 [0.22–3.75]		
40 to <50	14 (7.4)	22 (9.2)	0.046	2.83 [1.02–7.87]		
50 to <60	49 (26.01)	64 (26.7)	0.051	2.35 [1.00–5.54]		
60 to <70	56 (29.8)	71 (29.6)	0.057	2.28 [0.98–5.33]		
≥70	43 (22.9)	69 (28.8)	0.016	2.89 [1.22–6.84]		
Body mass index – Mean (kg/m^2^)	31.1 ± 6.4	31.8 ± 5.4	0.243^†^			
<18.5	7 (3.7)	6 (2.5)	0.222	1	0.594	NS
18.5 to <25	19 (10.1)	11 (4.6)	0.560	0.68 [0.18–2.53]		
25 to <30	48 (25.5)	67 (27.9)	0.407	1.63 [0.52–5.15]		
30 to <40	95 (50.5)	135 (56.3)	0.377	1.66 [0.54–5.09]		
≥40	12 (6.4)	15 (6.3)	0.578	1.46 [0.39–5.51]		
Missing data	7 (3.7)	6 (2.5)				
Race or ethnic group					
Non-Hispanic Black	11 (5.9)	9 (3.8)	0.570	1	0.341	NS
Hispanic	8 (4.3)	12 (5.0)	0.344	1.83 [0.52–6.43]	0.756	NS
Other	169 (89.9)	219 (91.3)	0.319	1.58 [0.64–3.91]		
Abdominal surgery history	14 (7.4)	34 (14.2)	0.031	1.76 [1.17–2.65]	0.051	NS
**Social history**
Smoking/tobacco use	60 (31.9)	90 (37.5)	0.230	1.28 [0.86–1.92]	0.982	NS
Alcohol use	68 (36.2)	122 (50.8)	0.003	1.83 [1.24–2.70]	0.006	1.74 (1.17–2.59)
Depressive disorder	37 (19.7)	79 (32.9)	0.002	2.03 [1.28–3.14]	0.005	1.90 (1.21–3.00)
Major depressive disorder	27 (14.4)	57 (23.8)	0.016	1.86 [1.12–3.08]	0.884	NS
**Coexisting conditions**
Incontinence	2 (1.1)	6 (2.5)	0.291	2.39 [0.48–11.95]	0.380	NS
Pelvic and perineal pain	16 (8.5)	27 (11.3)	0.351	1.36 [0.71–2.61]	0.620	NS
Strain of muscle, fascial and tendon	17 (9.0)	33 (13.8)	0.135	1.60 [0.86–2.98]	0.333	NS
Strain of muscle, fascial and tendon of lower back	3 (1.6)	4 (1.7)	0.954	1.05 [0.23–4.723]	0.603	NS
Low back pain	53 (28.2)	98 (40.8)	0.007	1.76 [1.17–2.65]	0.116	NS

*(1)^†^Wilcoxon W non-parametric Test; (2) NS, not significant*.

**Table 5:** Characteristics of RAD women with or without hernia.

**Variable**	**No. (%)**		**Univariate**		**Multivariate**	
	**Non-hernia subgroup (*N* = 566)**	**Hernia subgroup (*N* = 310)**	***P*-value**	**OR (95% CI)**	***P*-value**	**OR (95% CI)**
**DEMOGRAPHIC**
Age, mean (SD), y	40.6 ± 11.1	46.9 ± 13.8	0.000^†^			
<30	27 (4.9)	4 (1.3)	0.000	1	0.000	1.51 (1.33–1.72)
30 to <40	259 (46.6)	103 (33.2)	0.072	2.68 [0.92–7.86]		
40 to <50	198 (35.6)	111 (35.8)	0.015	3.78 [1.29–11.09]		
50 to <60	38 (6.8)	30 (9.7)	0.004	5.33 [1.68–16.90]		
60 to <70	21 (3.8)	33 (10.6)	0.000	10.61 [3.25–34.66]		
≥70	13 (2.3)	29 (9.4)	0.000	15.06 [4.37–51.89]		
Body mass index – Mean (kg/m^2^)	27.4 ± 6.3	29.2 ± 7.1	0.001^†^			
<18.5	10 (1.8)	4 (1.3)	0.001	1	0.235	NS
18.5 to <25	204 (36.7)	100 (32.3)	0.736	1.23 [0.38–4.00]		
25 to <30	182 (32.7)	81 (26.1)	0.860	1.11 [0.34–3.65]		
30 to <40	124 (22.3)	93 (30.0)	0.301	1.88 [0.57–6.17]		
≥40	21 (3.8)	28 (9.0)	0.067	3.33 [0.92–12.11]		
Missing data	15 (2.7)	4 (1.3)				
Race or ethnic group						
Non-Hispanic Black	23 (4.1)	19 (6.1)	0.427	1	0.128	NS
Hispanic	23 (4.1)	13 (4.2)	0.415	0.68 [0.28–1.70]	0.810	NS
Other	510 (91.7)	278 (89.7)	0.192	0.66 [0.35–1.23]		
Abdominal surgery history	175 (31.5)	90 (29.0)	0.455	0.89 [0.66–1.21]	0.590	NS
**Social history**
Smoking/tobacco use	69 (12.4)	72 (23.2)	0.000	2.14 [1.48–3.08]	0.010	1.66 (1.13–2.44)
Alcohol use	202 (36.6)	124 (40.0)	0.286	1.17 [0.88–1.55]	0.863	NS
Depressive disorder	127 (22.8)	96 (31.0)	0.009	1.52 [1.11–2.07]	0.223	NS
Major depressive disorder	86 (15.5)	66 (21.3)	0.031	1.48 [1.04–2.11]	0.307	NS
**Coexisting conditions**
Incontinence	20 (3.6)	15 (4.8)	0.375	1.36 [0.69–2.70]	0.861	NS
Pelvic and perineal pain	94 (16.9)	50 (16.1)	0.768	0.95 [0.65–1.38]	0.773	NS
Strain of muscle, fascial and tendon	10 (1.8)	4 (1.3)	0.571	0.71 [0.22–2.30]	0.181	NS
Strain of muscle, fascial and tendon of lower back	126 (22.7)	85 (27.4)	0.118	1.29 [0.94–1.77]	0.940	NS
Low back pain	175 (31.5)	90 (29.0)	0.455	0.89 [0.66–1.21]	0.590	NS

*(1)Wilcoxon W non-parametric Test; (2) NS, not significant*.

## Discussion

In this large multicenter retrospective study, significant gender differences with regards to RAD were demonstrated. Firstly, women had an approximately two-fold greater risk of developing RAD compared with men, and the age at onset in women was found to be two decades earlier than that in men. Secondly, men with RAD had higher BMI value than women with RAD. Thirdly, women with RAD underwent more than double the number of abdominal surgeries compared with men. In a previous study, we noted that the risk of RAD in women who underwent cesarean delivery (CD) was 4.7 times greater than those who delivered vaginally (18). This finding was consistent with the studies by Reinpold et al. (4), Werner and Dayan (19), and Hills et al. (20). It was also reported that pregnancy related to the number of pregnancies and multiple births increase the risk of developing RAD during the course of pregnancy (3). Given the characteristics mentioned above and the data presented in previous findings, it is reasonable to infer that pregnancy, delivery, and CD might be important factors leading to the development of RAD in female population, but the precise mechanism has not been well-recognized yet.

In our study, abdominal wall hernia was the most common complaint in patients with RAD, with rates higher than 40%. It was more common in men than in women. Approximately five million Americans have abdominal wall hernias, the majority of which are inguinal hernias (16, 21). This implies that RAD cases have over 20 times more abdominal hernia compared with the general population. The most common type of hernia in men was ventral hernia (35.8%), whereas umbilical hernia (33.8%) was the most common in women. Umbilical hernia in this set of subjects was a prominent condition with an over one-third occurrence, which was also higher than the rate of 23–25% in individuals, detected by high-resolution ultrasonography imaging (22). Therefore, in both men and women in the RAD population, the rate of abdominal wall hernia was significantly higher than that in the general population in USA, and the distribution of hernia type in RAD individuals differed from the general population as well. Interestingly, the cumulative composition ratio of hernia before 50 years of age was higher in women than in men, but, eventually, the men had a higher incidence of hernia. This might relate to that women had a pregnancy history and underwent more than double the number of abdominal surgeries than men before the age at onset of RAD.

Additionally, we demonstrated that smoking and age might be risk factors contributing to coexisting hernias in women with RAD. Smoking is a modifiable risk factor impacting outcomes in patients undergoing hernia repair and is the risk factor for the development of inguinal hernia in the general population (23, 24). Our study also confirmed that a woman with RAD who is a smoker/tobacco user is 1.66 times more likely to develop a concomitant hernia than those who are non-smokers/tobacco users. The development of hernia from smoking/tobacco use might be explained by the decrease of collagen synthesis and tissue oxygenation, which lead to lowering of the function of abdominal wall tissue repair, especially for those patients who undergo open abdominal wall surgery (25). Furthermore, for women with RAD, the risk of coexisting hernia increases 1.51 times with the age increasing per decade. This is consistent with a study that found the prevalence of femoral hernias increases steadily throughout life (26).

Alcohol use and depressive disorder contribute to the development of coexisting hernias in men with RAD. It was reported that men who take ≥20 g/day of alcohol are more likely to develop a hiatal hernia (27). We also confirmed this positive association between alcohol use and the occurrence of hernia in men with RAD; men with RAD who used alcohol have 1.74 times the risk of development of hernia than non-alcohol users. However, this is in contrast to some other studies (28, 29). Therefore, further prospective study with large samples on hernias is worth carrying out to identify whether alcohol use is associated with hernias. To our knowledge, it has never been reported about the relationship between depressive disorder and hernia. We found that over one-quarter subjects in our study had depressive disorder, and men with the depressive disorder had 1.90 greater risks of coexisting hernia than those without the depressive disorder in the RAD population. It was reported that physical pain symptoms are associated with some subjective feelings of depression (30). In our study, there were ~30% cases with low back pain and 15% cases with pelvic perineal pain in these RAD samples. Whether or not hernia, low back pain, pelvic and perineal pain, and depressive disorder interact with each other is unclear. This finding also suggests that further research to explore the underlying mechanisms of the association between depressive disorder, hernias, and chronic pain in patients with RAD are warranted.

Obesity has been reported as the risk factor for the development of hernia (16). In our study, women with BMI greater or equal to 30 kg/m^2^ indeed had a higher prevalence of hernia, including diaphragmatic hernia, ventral hernia, and incisional hernia compared with those whose BMI is <30 kg/m^2^. However, this phenomenon did not appear in the male group, and BMI as an independent variable failed to enter the multivariable binary logistic regression analysis model when using hernia as the dependent variable in both the male and the female group. Thus, in RAD population, the impact of obesity on the development of hernia deserves further evaluation.

Although RAD is not a hernia, the concomitant hernia is an important factor for RAD patients to seek medical attention. However, to date, it remains to be said if there is indeed a relationship between RAD and hernia and if surgical repair techniques to correct the hernia have any cosmetic or functional advantage in terms of the reinforcement of the ventral abdominal wall. Therefore, understanding the patterns and risk factors of hernia in patients with RAD is important, and further study of this issue is warranted as well.

## Limitations

This investigation has several limitations. Given the retrospective nature of the study, selection and measurement bias may have occurred. We also did not subcategorize the types of RAD due to the misinformation. Furthermore, we relied on ICD-9-CM and ICD-10-CM diagnosis codes for RAD and coexisting conditions, which could be under-diagnosed due to coding conditions and record keeping within the RPDR system. Nevertheless, we acknowledge the biases that could be introduced by the retrospective study. Fortunately, we are confident that the sample size of our study is reasonable for analysis.

## Conclusions

In conclusion, this study showed that the demographic characteristics, the prevalence and the risk factors of hernia in women with RAD significantly differed from that in men with RAD. Female subjects demonstrated a 1.9 times greater risk of developing RAD than male subjects, both mean age at onset of RAD and mean BMI in women were significantly lower than that in men. Over 40% of the subjects had one or more hernia, and it was more common in men with RAD than that in women. Umbilical hernia is an important type of hernia with one-third occurrence in this population. Alcohol use and depressive disorder in men and age and smoking in women were risk factors of coexisting hernias in patients with RAD.

## Data Availability Statement

The original contributions presented in the study are included in the article, further inquiries can be directed to the corresponding author.

## Ethics Statement

The studies involving human participants were reviewed and approved by Partners' institutional review board of the Research. Written informed consent for participation was not required for this study in accordance with the national legislation and the institutional requirements.

## Author Contributions

SY helped to develop ideas for study, collected the data, analyzed the data, and prepared the manuscript draft. HW helped to develop ideas for study and reviewed the manuscript. JZ contributed to development of ideas, the study design, and manuscript preparation for publication. All authors contributed to the article and approved the submitted version.

## Conflict of Interest

The authors declare that the research was conducted in the absence of any commercial or financial relationships that could be construed as a potential conflict of interest.

## Publisher's Note

All claims expressed in this article are solely those of the authors and do not necessarily represent those of their affiliated organizations, or those of the publisher, the editors and the reviewers. Any product that may be evaluated in this article, or claim that may be made by its manufacturer, is not guaranteed or endorsed by the publisher.
